# Fluoroquinolones and Other Antibiotics Redeemed for Cystitis—A Swedish Nationwide Cohort Follow-Up Study (2006–2018)

**DOI:** 10.3390/antibiotics11020172

**Published:** 2022-01-28

**Authors:** Xinjun Li, Kristina Sundquist, Filip Jansåker

**Affiliations:** 1 Center for Primary Health Care Research, Department of Clinical Sciences Malmö, Lund University, 205 02 Malmö, Sweden; xinjun.li@med.lu.se (X.L.); kristina.sundquist@med.lu.se (K.S.); 2 Center for Community-Based Healthcare Research and Education (CoHRE), Department of Functional Pathology, School of Medicine, Shimane University, Matsue 690-0823, Japan; 3 Department of Family Medicine and Community Health, Department of Population Health Science and Policy, Icahn School of Medicine at Mount Sinai, New York, NY 10029, USA

**Keywords:** antibiotics, cystitis, fluoroquinolones

## Abstract

Background: Antibiotics are commonly prescribed for outpatient management of cystitis. Previous evidence suggests that certain factors likely beyond the infection seem to influence the choice of antimicrobial treatment. However, studies on the specific antibiotic treatments for cystitis are lacking. This study aimed to explore the antibiotic treatments for cystitis using nationwide primary healthcare data and investigate if factors beyond the infection could be associated with fluoroquinolone treatment. Methods: This nationwide follow-up cohort study consisted of 352,507 women with cystitis. The primary aim was to investigate what specific classes of antibiotics were redeemed by patients within five days from the cystitis diagnosis. Each patient could only be included once. Logistic regression models were also used to examine the relationship between fluoroquinolone (FQ) treatment, parity, and sociodemographic factors. Results: In total, 192,065 antibiotic prescriptions were redeemed. Pivmecillinam (58.4%) followed by nitrofurantoin (22.2%), trimethoprim (12.0%), fluoroquinolone (5.6%), and cephalosporins (1.5%) were the most redeemed antibiotics. Sociodemographic factors were weakly associated with fluoroquinolone treatment; young age was inversely associated with fluoroquinolone treatment. Parity and cervical cancer history were not associated with fluoroquinolone treatment. The proportion of fluoroquinolone and trimethoprim treatments decreased over time, while pivmecillinam and nitrofurantoin increased. Conclusions: The treatment trends of antibiotics redeemed within five days from a cystitis diagnosis were similar to the national surveillance program of these antibiotics (not diagnosis linked). Fluoroquinolones were weakly associated with sociodemographic factors, which likely is only of historical relevance.

## 1. Introduction

Uncomplicated lower urinary tract infection (UTI), also known as uncomplicated cystitis, is one of the most common bacterial infections in women. About one in ten women have at least one infection each year and therefore cystitis is an important contributor to antibiotics exposure in otherwise healthy women in Sweden and worldwide [1,2,3,4,5].

However, over the last decades, safe and effective treatment options have decreased. This is partly due to an increase in antibiotic resistance, making certain antibiotics, such as fluoroquinolones, less suitable as empirical treatment options for cystitis. For example, fluoroquinolone resistance has passed 20% in several countries worldwide. Furthermore, certain UTI antibiotics have also been found to be more associated with collateral damage and severe adverse effects. For example, suspected cardiotoxicity and neuropsychiatric conditions have been related to fluoroquinolones [6,7,8,9,10,11,12,13,14,15,16,17,18,19,20].

All this considered, the treatment guidelines for cystitis (including in Sweden) have turned away from certain antibiotics—mainly fluoroquinolones, reserved for more serious infections—towards more narrow-spectrum antibiotics, such as nitrofurantoin and pivmecillinam, with lower resistance rates and more suitable safety profiles for use in the treatment of cystitis [8,19,21]. The national antibiotic surveillance program has demonstrated this shift [22], which has also been seen in smaller local but diagnosis-linked studies [23,24]. However, no study has investigated on a population-based dataset UTI treatment trends for a diagnosis of uncomplicated cystitis, nor how large of a proportion of certain antibiotics (e.g., fluroquinolones) comprise and if this could vary due to factors beyond the infection.

The gap in previous research is most likely due to the lack of nationwide population-based data from primary healthcare, where UTIs are mostly managed. We recently identified that about 54.5% of women diagnosed with uncomplicated cystitis in Sweden redeemed an antibiotic for their prescription. Furthermore, the study found that certain factors (likely beyond the infection) were associated with antibiotic treatment for cystitis [4]. In this follow-up study, by using a similar method [4], we aimed to study what specific classes of antibiotics were redeemed for cystitis in Sweden over a 13-year time period and determine if the annual trends were similar to the national surveillance program of antibiotics. We also intended to study if factors likely beyond the infection (e.g., history of cervical cancer and parity, and sociodemographic factors) could be associated with fluoroquinolone treatment.

## 2. Results

Table 1 includes the annual proportional distribution of each of the investigated antibiotic groups amongst the 192,065 antibiotic treatments for uncomplicated cystitis during the study period. The table shows that penicillins with extended spectrum (pivmecillinam) were used in 58.4% of all cases, followed by nitrofurantoin (22.2%), trimethoprim (12.0%), fluoroquinolones (5.6%), cephalosporins (1.5%), sulphonamides/trimethoprim combinations (0.4%), and beta-lactam/beta-lactamase inhibitors (<0.1%). During the first couple of years, treatment with fluoroquinolones, cephalosporins, and especially trimethoprim was more common but declined prominently in the following years. Nitrofurantoin was not widely used in our population during the first years, but as fluoroquinolones and trimethoprim use decreased, nitrofurantoin treatment increased. From 2011–2018, nitrofurantoin was used in about one-third of all cases. Pivmecillinam was used in about 60% of all cases annually (except for the first two years). Supplementary Table S1 includes data on when seven-level ATC codes from 2006 to 2013 were available. Pivmecillinam (J01CA08) accounted for about 57.4% of all these, followed by nitrofurantoin (20.1%), trimethoprim (13.6%), and fluoroquinolones (5.9%). Pivmecillinam also accounted for 99.4% of the penicillin with extended spectrum (amoxicillin, J01CA04, accounted for the other 0.6%). The proportion of nitrofurantoin and pivmecillinam treatments also seemed to increase in parallel to the decline of trimethoprim and fluoroquinolone treatments.

Table 2 presents a fully adjusted model of the associations between the individual sociodemographic variables and fluoroquinolone treatment within five days after the uncomplicated cystitis event. Age and education level were weakly associated with fluoroquinolone treatment. For example, middle followed by low education level had higher odds compared to high education level. Low income was associated with an increased odds of fluoroquinolone treatment. For example, the lowest income quartile had an OR of 1.30 (95% CI 1.23–1.38) compared to the highest income quartile. Living outside larger cities was strongly associated with lower odds of fluoroquinolone treatment. Country of origin did not seem to have a strong association with fluoroquinolone treatment in general. However, women from Western countries, Asia (excluding the Middle East) and Oceania, and Latin America and the Caribbean seemed to have significantly increased odds of fluoroquinolone treatment compared to Swedish women. History of cervical cancer and parity were associated with a decreased odds of fluoroquinolone treatment, however, only to a statistically significant extent for the latter. Year of infection (continuously increasing) was significantly associated with a decrease in fluoroquinolone treatment.

## 3. Discussion

This study presents diagnosis-linked antibiotic treatment trends for uncomplicated cystitis on a nationwide level. As expected, pivmecillinam and nitrofurantoin were the two most used antibiotics. The annual proportion of these antibiotics increased over time, as the proportion of trimethoprim and fluoroquinolones decreased. Year of infection (continuously increasing) was also inversely associated with fluoroquinolone treatment rates. These trends were in accordance with the national guidelines [21] and the general trend of UTI antibiotics (not linked to diagnoses) in women in the national surveillance program over the same time period [25]. Furthermore, annual treatment trends were comparable to that of smaller local studies [23,24]. The consistencies support our findings, and altogether this indicates that the national surveillance program of antibiotics [25] seems to be representative and valid for cystitis. These findings could represent important information for healthcare planners working on national antibiotic surveillance and for the clinical implications of these programs for this very common bacterial infection.

Less than six percent of all the antibiotic treatments linked to uncomplicated cystitis were fluoroquinolones (mainly ciprofloxacin). Factors likely beyond the infection—such as sociodemographic factors and history of cervical cancer and parity—did not seem to be associated with fluoroquinolone treatment for uncomplicated cystitis to a high extent. Women from low family income backgrounds seemed to have received more fluoroquinolone treatments than women of higher socioeconomic status. For country of origin, only MENA women had lower odds of fluoroquinolone compared to Swedish-born women (although statistically non-significant), while women from Asia (excluding Middle East) and Oceania, Western countries, and Latin America and the Caribbean had weak but significantly higher odds of fluoroquinolone treatment compared to Swedish-born women. The mechanisms behind these sociodemographic differences in fluoroquinolone treatment of uncomplicated cystitis, which were similar to the odds for antibiotic treatment in general [4], could, inter alia, be attributed to uncertainty in diagnostic testing [18]. During the last decade, it has not been recommended to prescribe fluoroquinolones as a first-line prescription for uncomplicated cystitis [19], as these antibiotics ought to be limited to serious infections and not uncomplicated cystitis due to concern of worrisome collateral damage and adverse effects associated with these antibiotics [15,16,17,18,19,20]. If the sociodemographic differences associated with fluoroquinolones still exist, it would be of particular concern. However, fluoroquinolones were not included in the recent Swedish guidelines for cystitis [21], and the prescription of these drugs has in general been drastically declining over the recent years [25], which was also seen in our study with diagnosis-linked data. Hence, it is possible that these findings are of more historic relevance in Sweden.

Limitations of this study were that we did not have access to data on the symptoms or clinical presentation in the patients, nor did we have data on adherence rates. Therefore, no firm conclusion can be made on whether the differences in fluoroquinolone treatment could have been due to unequal healthcare (differences in prescription rates) or differences in adherence (differences in pick-up rates). Further studies could examine possible mechanisms behind the found associations of factors likely beyond the infection and antibiotic treatments [4]. The main strengths of this study include that this study involved several highly validated nationwide patient and population data registries including nationwide primary healthcare data. Previous population-based studies on antibiotic treatments of primary healthcare infections have, to the best of our knowledge, not included data of diagnoses linked to antibiotic prescriptions.

Antibiotics are important drugs for patients worldwide [26] and should be given when needed. However, due to the growing threat of antimicrobial resistance [10,13,14] and potential severe adverse reactions [15,16,17,18,19,20], certain drugs, such as fluoroquinolones, ought to be saved for more serious infections. This study demonstrates that the shift in treatment recommendations for uncomplicated cystitis seems to have been followed. In addition to this, the findings support that the national surveillance program of antibiotics, although lacking the diagnoses linked to the antibiotics, likely is representative, at least for UTI antibiotics in women. Further population-based diagnosis-linked studies using primary healthcare data are needed to validate these findings on other infections before a general conclusion can be made.

## 4. Materials and Methods

### 4.1. Study Design

This was a population-based cohort register study and follow-up to a recent nationwide study of ours [4]. The time period was from 1 January 2006 to 31 December 2018. The study used several national registers together with nationwide primary healthcare data.

### 4.2. Setting

This research was conducted at the Center for Primary Health Care Research at Lund University and Region Skåne, Malmö, Sweden. The STROBE statement for cohort studies was considered when conducting this study and in the writing process [27].

### 4.3. Study Population

The study population consisted of 352,507 women [4] between the age of 15 and 50 years at the time of being diagnosed with a first event of uncomplicated cystitis during the study period. The 10th revision of the International Classification of Diseases (ICD-10) was used to identify the code N30 for acute infective cystitis [5]. Cases with diagnoses not considered to be in accordance with uncomplicated cystitis were not included in the study: i.e., ICD-10 codes of N301-304, and N308, or co-morbidities (ICD-10: B20-24, C64-68, D41, D80-89, E10-11, M623, N03, N07, N11, N13-23, N25-29, N32, Q60-64) diagnosed within two years prior to the cystitis episode. Women with ongoing pregnancy or treatment with immunomodulating agents were also not considered [4]. Each woman could only be included once.

### 4.4. Outcome Variable

The outcome variable (redeemed antibiotic prescription) was defined as the first UTI antibiotic redeemed (measured as prescription from a physician dispensed at a pharmacy) within five days after a diagnosis of uncomplicated cystitis. The outcome is generally referred to as “antibiotic treatment” in the manuscript. The following antibiotic groups were assessed based on their ATC code: penicillins with extended spectrum (J01CA), for example, pivmecillinam; beta-lactam/b-lactam inhibitor combinations (J01CR); cephalosporins (J01DB-E,I); trimethoprim derivatives (J01EA), i.e., trimethoprim; sulphonamides/trimethoprim combinations (J01EE), i.e., trimethoprim/sulphamethoxazole; fluoroquinolones (J01MA), for example ciprofloxacin; and nitrofuran derivatives, i.e., nitrofurantoin (J01XE). Seven-level ATC codes were available during the first eight years (2006–2013) of the study. Oral drugs were exclusively assessed. Mono-sulphonamide antibiotics and fosfomycin were not considered, as these antibiotics were not widely available in Sweden at the time of the study. We did not consider either antibiotics that were not recommended [19,21] to be used or generally prescribed for patients with cystitis (e.g., tetracyclines, macrolides).

### 4.5. Predictor Variables for Fluoroquinolone Treatment

The predictors investigated were measured at baseline (i.e., time of the cystitis diagnosis). Age groups were defined as being between 15–24, 25–34, 35–44, or 45–50 years of age. Country of origin was categorized as originating from any of the following countries/regions: Sweden; Eastern European countries; Western countries; Middle East/North Africa (MENA); Africa (excluding North Africa); Asia (excluding Middle East) and Oceania; or Latin America/the Caribbean. Countries with geographical proximity and/or cultural and economic similarities were categorized together. Both first- and second-generation immigrants were included in country groups other than Sweden. The categories for this study were based on the definition used in a previous study of ours [5]. Educational level was classified into three different categories based on the duration of school years attended: compulsory schooling or less (≤9 years); short or partially completed high school education (10–11 years); or completed high school education or more, such as university or college education (≥12 years). For those aged 15–17 years in the youngest age group, the highest educational level of the parents was used. Family income was categorized into four groups based on a weighted average income in each family: low (lowest income quartile of the study population), middle low/middle high (second/third quartiles), and high (highest quartile). Region of residence was grouped as large cities, Southern Sweden, and Northern Sweden. Parity was defined from the Swedish Medical Birth Register and categorized as no child (nullipara) or at least one child. Cervical cancer (Yes/No) was defined from the Swedish Cancer Register according to ICD-7 code 171. As the prescription rate of fluoroquinolone decreased in Sweden [25] during the time period, the year of infection was included as a continuous (increasing) variable in the analysis.

### 4.6. Data Sources

This cohort study population was identified through population-based primary healthcare data (1997–2018) accessed from 21 out of 22 administrative regions in Sweden during the study period. The coverage of these data varied by time and region based on when the patient records were digitalized. The database included around 72% of the population in Sweden in 2015 and around 90% of the population at the end of the study. The following nationwide registers, managed by the National Board of Health and Welfare (in Swedish: *Socialstyrelsen*), were used to identify the outcome, parity, cervical cancer, as well as comorbidities or other complicating factors not aligned with uncomplicated cystitis: [1,28] the Swedish Prescribed Drug Register (2005–2018), which contains the specific ATC codes on redeemed drug prescriptions from all pharmacies in Sweden; the Medical Birth Register (1973–2018); the National Patient Register (including inpatient (1964–2018) and outpatient data (2001–2018)); the Cause of Death Register (1961–2018); and the Swedish Cancer Register (1958–2018). The Total Population Register (1968–2018) was used to collect data on age, country of origin, education, income, and region of residence. All linkages between the individual-level data in the databases were performed using a pseudonymized (encrypted) version of the unique 10-digit personal identification number (assigned to each person residing in Sweden during their lifetime).

### 4.7. Statistical Analysis

Descriptive statistics on the annual treatment trends (redeemed antibiotic prescription five days after a diagnosis of uncomplicated cystitis) were calculated for each antibiotic based on the five-level-ATC-code antibiotic group for the whole study period (Table 1). During the years when seven-level-ATC-code were available, a sub-analysis of specific antibiotic treatment proportions was conducted (Supplementary Material Table S1). To test for the association between the predictor variables and fluoroquinolone treatment, adjusted logistic regression models (Table 2) were used to estimate odds ratios (ORs) and 95% confidence intervals (CIs). The study period started on 1 January 2006 and proceeded until redeemed antibiotic prescription (within five days), death, emigration, or the end of the study period on 31 December 2018. Missing values (range 0.0–1.3%) were not excluded. A two-tailed *p*-value of <0.05 was used to define statistical significance. SAS version 9.4 (SAS Institute Inc.; Cary, NC, USA) was used for all statistical analyses.

### 4.8. Ethical Consideration

The present study was a non-intervention nationwide register study of pseudonymized secondary data obtained from Swedish authorities and was approved by the Ethical Review Board in Lund. All methods were performed in accordance with the relevant guidelines and regulations.

### 4.9. Role of Funding Source

The funding sources of this study were all non-commercial and had no role in the study design; the collection, analysis, and interpretation of data; the writing of the report; or the decision to submit the paper for publication.

## 5. Conclusions

The treatment trends of antibiotics (for urinary tract infections) redeemed within five days from a cystitis diagnosis were similar to the national surveillance program of these antibiotics (not diagnosis linked). The decreasing treatment rate of fluroquinolones over time was also in accordance with the national surveillance program. This indicates that the national surveillance program is likely representative and valid in identifying antibiotics treatments for urinary tract infections. Fluoroquinolone treatment was weakly associated with some sociodemographic factors and parity, which likely are beyond the infection but only of historical relevance, as these antibiotics seem to be used less for this infection in Sweden, in accordance with national guidelines. These findings represent important new information, particularly for healthcare planners involved with the national antibiotic surveillance of this very common bacterial infection in women.

## Figures and Tables

**Table 1:** Treatment trends of the 192,065 antibiotics redeemed for uncomplicated cystitis (2006–2018).

Antibiotic Groups in Order of Total Proportion (%)	Year													
	2006	2007	2008	2009	2010	2011	2012	2013	2014	2015	2016	2017	2018	All
Penicillins with extended spectrum (J01CA)	46.6	51.8	62.6	62.3	60.6	61.6	61.5	63.5	62.6	64.5	62.1	59.2	59.2	58.4
Nitrofuran derivatives (J01XE)	10.6	14.2	13.2	21.7	26.8	29.0	30.9	29.5	31.8	29.5	33.0	35.3	35.1	22.2
Trimethoprim and derivatives (J01EA)	28.0	22.1	16.2	10.5	7.7	5.2	3.5	2.7	1.7	1.6	0.9	0.8	0.5	12.0
Fluoroquinolones (J01MA)	11.4	9.0	5.6	4.1	3.7	3.1	2.9	3.3	3.0	3.5	2.9	3.7	4.1	5.6
Cephalosporins (J01DB-E,I)	3.0	2.5	2.0	1.2	0.9	0.8	0.9	0.8	0.7	0.7	1.0	0.5	0.7	1.5
Sulphonamides/Trimethoprim combinations (J01EE)	0.5	0.4	0.5	0.3	0.4	0.4	0.3	0.2	0.2	0.2	0.1	0.4	0.4	0.4
	100.0	100.0	100.0	100.0	100.0	100.0	100.0	100.0	100.0	100.0	100.0	100.0	100.0	100.0

No sulphonamide antibiotics (J01EB, J01EC, J01ED) were identified. Less than 0.1% of the antibiotic treatments were a beta-lactam/b-lactam inhibitor combination (J01CR, i.e., amoxicillin/clavulanic acid), hence why it is not part of the table. During 2006–2013, when seven-level ATC coding was available, in regard to J01CA, >99% was pivmecillinam and the remaining was amoxicillin; J01XE was nitrofurantoin; J01EA was trimethoprim; J01MA was ciprofloxacin (62%) and norfloxacin (38%). No sulphonamide antibiotics (J01EB, J01EC, J01ED) or fosfomycin (J01XX01) were identified. Each patient could only be included once during the study period. Data is from the Swedish Prescribed Drug Register.

**Table 2:** The association between history of cervical cancer, parity, and individual sociodemographic factors and fluoroquinolone treatment (10,684 cases) for uncomplicated cystitis.

Covariates	* OR	95% CI		*p*-Value
**Gynecological history** (ref. no history)				
Parity	0.92	0.87	0.96	<0.001
Cervical cancer	0.92	0.83	1.02	0.126
**Age** (ref. age 45–50 years)				
15–24	1.02	0.95	1.09	0.674
25–34	0.88	0.83	0.94	<0.001
35–44	0.95	0.90	1.01	0.082
**Educational level** (ref. ≥ 12 years)				
≤9	1.06	1.00	1.12	0.060
10–11	1.12	1.06	1.18	<0.001
**Family income** (ref. high)				
Low	1.30	1.23	1.38	<0.001
Middle-low	1.19	1.13	1.26	<0.001
Middle-high	1.07	1.01	1.13	0.019
**Region of residence** (ref. large cities)				
Southern Sweden	0.88	0.83	0.92	<0.001
Northern Sweden	0.84	0.78	0.91	<0.001
**Country of origin** (ref. Sweden)				
Eastern Europe	1.05	0.97	1.14	0.246
Western countries	1.21	1.10	1.33	<0.001
Middle East/North Africa	0.94	0.86	1.02	0.112
Africa (excluding North Africa)	1.05	0.92	1.19	0.502
Asia (excluding Middle East) and Oceania	1.25	1.13	1.37	<0.001
Latin America and the Caribbean	1.19	1.04	1.36	0.013
**Year of infection** (increasing)	0.93	0.92	0.93	<0.001

CI: confidence interval. OR: odds ratio. * Fully adjusted for all covariates. Time period: 2006–2018.

## Data Availability

This study made use of several national registers and, owing to legal concerns, data cannot be made openly available. Further information regarding the health registries is available from the Swedish National Board of Health and Welfare: https://www.socialstyrelsen.se/en/statistics-and-data/registers/ (accessed on 26 October 2021), and Kristina Sundquist.

## Supplementary Materials

The following are available online at https://www.mdpi.com/article/10.3390/antibiotics11020172/s1, Table S1: Number of specific UTI antibiotics in the four most common antibiotic treatment groups for uncomplicated cystitis (2006–2013).

## Abbreviations

ATC	Anatomic Therapeutic Chemical classification system
CI	Confidence interval
ICD	International Classification of Diseases
MENA	Middle East/North Africa
OR	Odds ratio
UTI	Urinary tract infection

## References

[1] Nicolle L.E. (2008). Uncomplicated urinary tract infection in adults including uncomplicated pyelonephritis. Urol. Clin. N. Am..

[2] Foxman B., Barlow R., D’Arcy H., Gillespie B., Sobel J.D. (2000). Urinary tract infection: Self-reported incidence and associated costs. Ann. Epidemiol..

[3] Butler C.C., Hawking M.K., Quigley A., McNulty C.A. (2015). Incidence, severity, help seeking, and management of uncomplicated urinary tract infection: A population-based survey. Br. J. Gen. Pract..

[4] Jansåker F., Li X., Knudsen J.D., Milos Nymberg V., Sundquist K. (2021). The Effect of Sociodemographic Factors, Parity and Cervical Cancer on Antibiotic Treatment for Uncomplicated Cystitis in Women: A Nationwide Cohort Study. Antibiotics.

[5] Jansåker F., Li X., Sundquist K. (2021). Sociodemographic factors and uncomplicated cystitis in women aged 15–50 years: A nationwide Swedish cohort registry study (1997–2018). Lancet Reg. Health-Eur..

[6] Schito G.C., Naber K.G., Botto H., Palou J., Mazzei T., Gualco L., Marchese A. (2009). The ARESC study: An international survey on the antimicrobial resistance of pathogens involved in uncomplicated urinary tract infections. Int. J. Antimicrob. Agents.

[7] Kahlmeter G., Poulsen H.O. (2012). Antimicrobial susceptibility of Escherichia coli from community-acquired urinary tract infections in Europe: The ECO.SENS study revisited. Int. J. Antimicrob. Agents.

[8] Poulsen H.O., Johansson A., Granholm S., Kahlmeter G., Sundqvist M. (2013). High genetic diversity of nitrofurantoin- or mecillinam-resistant Escherichia coli indicates low propensity for clonal spread. J. Antimicrob. Chemother..

[9] Kahlmeter G., Ahman J., Matuschek E. (2015). Antimicrobial Resistance of Escherichia coli Causing Uncomplicated Urinary Tract Infections: A European Update for 2014 and Comparison with 2000 and 2008. Infect. Dis. Ther..

[10] Holmes A.H., Moore L.S., Sundsfjord A., Steinbakk M., Regmi S., Karkey A., Guerin P.J., Piddock L.J. (2016). Understanding the mechanisms and drivers of antimicrobial resistance. Lancet.

[11] Abdelrady A.M., Zaitone S.A., Farag N.E., Fawzy M.S., Moustafa Y.M. (2017). Cardiotoxic effect of levofloxacin and ciprofloxacin in rats with/without acute myocardial infarction: Impact on cardiac rhythm and cardiac expression of Kv4.3, Kv1.2 and Nav1.5 channels. Biomed. Pharmacother..

[12] Sellick J., Mergenhagen K., Morris L., Feuz L., Horey A., Risbood V., Wojciechowski A., Ruh C., Bednarczyk E., Conway E. (2018). Fluoroquinolone-Related Neuropsychiatric Events in Hospitalized Veterans. Psychosomatics.

[13] Laxminarayan R., Van Boeckel T., Frost I., Kariuki S., Khan E.A., Limmathurotsakul D., Larsson D.G.J., Levy-Hara G., Mendelson M., Outterson K. (2020). The Lancet Infectious Diseases Commission on antimicrobial resistance: 6 years later. Lancet Infect. Dis..

[14] Roberts S.C., Zembower T.R. (2021). Global increases in antibiotic consumption: A concerning trend for WHO targets. Lancet Infect. Dis..

[15] Kalghatgi S., Spina C.S., Costello J.C., Liesa M., Morones-Ramirez J.R., Slomovic S., Molina A., Shirihai O.S., Collins J.J. (2013). Bactericidal antibiotics induce mitochondrial dysfunction and oxidative damage in Mammalian cells. Sci. Transl. Med..

[16] Kaur K., Fayad R., Saxena A., Frizzell N., Chanda A., Das S., Chatterjee S., Hegde S., Baliga M.S., Ponemone V. (2016). Fluoroquinolone-related neuropsychiatric and mitochondrial toxicity: A collaborative investigation by scientists and members of a social network. J. Community Support. Oncol..

[17] Kuula L.S.M., Viljemaa K.M., Backman J.T., Blom M. (2019). Fluoroquinolone-related adverse events resulting in health service use and costs: A systematic review. PLoS ONE.

[18] Redgrave L.S., Sutton S.B., Webber M.A., Piddock L.J. (2014). Fluoroquinolone resistance: Mechanisms, impact on bacteria, and role in evolutionary success. Trends Microbiol..

[19] Gupta K., Hooton T.M., Naber K.G., Wullt B., Colgan R., Miller L.G., Moran G.J., Nicolle L.E., Raz R., Schaeffer A.J. (2011). International clinical practice guidelines for the treatment of acute uncomplicated cystitis and pyelonephritis in women: A 2010 update by the Infectious Diseases Society of America and the European Society for Microbiology and Infectious Diseases. Clin. Infect. Dis..

[20] Mathews B., Thalody A.A., Miraj S.S., Kunhikatta V., Rao M., Saravu K. (2019). Adverse Effects of Fluoroquinolones: A Retrospective Cohort Study in a South Indian Tertiary Healthcare Facility. Antibiotics.

[21] (2017). Drug Treatment of Urinary Tract Infections in Outpatient Care—Treatment Recommendation: Information from the Swedish Medical Products Agency. [Läkemedelsbehandling av Urinvägsinfektioner i Öppenvård—Behandlingsrekommendation: Information från Läkemedelsverket].

[22] Ternhag A., Grunewald M., Naucler P., Wisell K.T. (2014). Antibiotic consumption in relation to socio-demographic factors, co-morbidity, and accessibility of primary health care. Scand. J. Infect. Dis..

[23] Lindback H., Lindback J., Melhus A. (2017). Inadequate adherence to Swedish guidelines for uncomplicated lower urinary tract infections among adults in general practice. APMIS.

[24] Kornfalt Isberg H., Melander E., Hedin K., Molstad S., Beckman A. (2019). Uncomplicated urinary tract infections in Swedish primary care; etiology, resistance and treatment. BMC Infect. Dis..

[25] (2019). SWEDRES-SVARM 2019. Consumption of Antibiotics and Occurrence of Resistance in Sweden.

[26] Adedeji W.A. (2016). The Treasure Called Antibiotics. Ann. Ib. Postgrad. Med..

[27] Von Elm E., Altman D.G., Egger M., Pocock S.J., Gotzsche P.C., Vandenbroucke J.P., Initiative S. (2007). The Strengthening the Reporting of Observational Studies in Epidemiology (STROBE) statement: Guidelines for reporting observational studies. Lancet.

[28] Sabih A., Leslie S.W. (2020). Complicated Urinary Tract Infections. StatPearls.

