# Supplementary material for: Fluoroquinolones and Other Antibiotics Redeemed for Cystitis—A Swedish Nationwide Cohort Follow-Up Study (2006–2018)
Source: Antibiotics (Basel). 2022 Jan 28;11(2):172. doi: 10.3390/antibiotics11020172 (PMC8868163; doi:10.3390/antibiotics11020172)
Supplement: Supplementary file 1 [file antibiotics-11-00172-s001.zip › antibiotics-1530648-supplementary.pdf]

Supplementary Material

Table S1. Number of specific UTI antibiotics in the four most common antibiotic treatment groups for uncomplicated cystitis (2006–2013)

Groups	Specific UTI antibiotics in each group	**2006**	**2007**	**2008**	**2009**	**2010**	**2011**	**2012**	**2013**	All
J01CA	**Amoxicillin** (J01CA04)	133	111	78	59	45	51	39	34	550
	**Pivmecillinam** (J01CA08)	13 437	12 530	13 229	12 255	12 206	11 554	10 267	9768	95 246
J01XE	**Nitrofurantoin** (J01XE01)	3093	3473	2815	4283	5411	5465	5177	4559	34 276
J01EA	**Trimethoprim** (J01EA01)	8150	5386	3438	2079	1556	974	591	416	22 590
J01MA	**Ciprofloxacin** (J01MA02)	1171	1081	830	727	675	546	466	498	5994
	**Norfloxacin** (J01MA06)	2128	1102	351	83	72	36	15	6	3793
	All UTI specific antibiotics	29 146	24 406	21265	19 780	20 215	18 853	16 769	15 437	165 871

Each case represents one patient only. UTI=Urinary tract infection. Cephalosporins (J01DB-E,I), Sulphonamides/Trimethoprim combinations (J01EE), and beta-lactam/b-lactam inhibitor combination (J01CR) were too few to be included. No sulphonamide antibiotics (J01EB, J01EC, J01ED) or fosfomycin (J01XX01) were identified. Each patient could only be included once during the study period. Data from the Swedish Prescribed Drug Register.
